# Cerebellar White Matter Abnormalities in Charcot–Marie–Tooth Disease: A Combined Volumetry and Diffusion Tensor Imaging Analysis

**DOI:** 10.3390/jcm10214945

**Published:** 2021-10-26

**Authors:** Sungeun Hwang, Chang-Hyun Park, Regina Eun-Young Kim, Hyeon Jin Kim, Yun Seo Choi, Sol-Ah Kim, Jeong Hyun Yoo, Ki Wha Chung, Byung-Ok Choi, Hyang Woon Lee

**Affiliations:** 1 Departments of Neurology, Ewha Womans University Mokdong Hospital, Seoul 07985, Korea; neurosung@gmail.com; 2 Departments of Neurology, Ewha Womans University School of Medicine and Ewha Medical Research Institute, Seoul 07985, Korea; park.changhyun@hanmail.net (C.-H.P.); hkimneurology@gmail.com (H.J.K.); 3 Institute for Human Genomic Study, College of Medicine, Korea University, Ansan 15355, Korea; eunyoung.regina.kim@gmail.com; 4 Department of Psychiatry, University of Iowa, Iowa City, IA 52242, USA; 5 Department of Neurology, Korea University Ansan Hospital, Ansan 15355, Korea; 6 Departments of Medical Science, Ewha Womans University School of Medicine and Ewha Medical Research Institute, Seoul 07804, Korea; coolgirldew@naver.com (Y.S.C.); solak1221@naver.com (S.-A.K.); 7 Department of Radiology, College of Medicine, Ewha Womans University, Seoul 07985, Korea; yoolee@ewha.ac.kr; 8 Department of Biological Sciences, Kongju National University, Kongju 32588, Korea; kwchung@kongju.ac.kr; 9 Department of Neurology, Samsung Medical Center, Sungkyunkwan University School of Medicine, Seoul 06351, Korea; 10 Department of Health Sciences and Technology, Samsung Advanced Institute for Health Sciences & Technology, Sungkyunkwan University, Seoul 06351, Korea; 11 Stem Cell & Regenerative Medicine Institute, Samsung Medical Center, Seoul 06351, Korea; 12 Department of Computational Medicine, Ewha Womans University, Seoul 07985, Korea; 13 Department of System Health Science & Engineering, Ewha Womans University, Seoul 03765, Korea

**Keywords:** Charcot–Marie–Tooth disease (CMT), volumetry, diffusion tensor imaging (DTI), white matter, cerebellum, ataxia

## Abstract

Charcot–Marie–Tooth disease (CMT) is a genetically heterogeneous hereditary peripheral neuropathy. Brain volumetry and diffusion tensor imaging (DTI) were performed in 47 controls and 47 CMT patients with *PMP22* duplication (*n* = 10), *MFN2* (*n* = 15), *GJB1* (*n* = 11), or *NEFL* mutations (*n* = 11) to investigate for structural changes in the cerebellum. Volume of cerebellar white matter (WM) was significantly reduced in CMT patients with *NEFL* mutations. Abnormal DTI findings were observed in the superior, middle, and inferior cerebellar peduncles, predominantly in *NEFL* mutations and partly in *GJB1* mutations. Cerebellar ataxia was more prevalent in the *NEFL* mutation group (72.7%) than the *GJB1* mutation group (9.1%) but was not observed in other genotypic subtypes, which indicates that structural cerebellar abnormalities were associated with the presence of cerebellar ataxia. However, *NEFL* and *GJB1* mutations did not affect cerebellar gray matter (GM), and neither cerebellar GM nor WM abnormalities were observed in the *PMP22* duplication or *MFN2* mutation groups. We found structural evidence of cerebellar WM abnormalities in CMT patients with *NEFL* and *GJB1* mutations and an association between cerebellar WM involvement and cerebellar ataxia in these genetic subtypes, especially in the *NEFL* subgroup. Therefore, we suggest that neuroimaging, such as MRI volumetry or DTI, for CMT patients could play an important role in detecting abnormalities of cerebellar WM.

## 1. Introduction

Charcot–Marie–Tooth disease (CMT) is a hereditary peripheral neuropathy with clinical and genetic heterogeneities [1]. Peripheral myelin protein 22 (*PMP22*) duplication is known to cause CMT1A, while mitofusin 2 (*MFN2*) mutations cause CMT2A and gap junction protein 1 (*GJB1*) mutations are relevant to CMTX1 [2]. Mutations in the neurofilament light chain polypeptide (*NEFL*) produce a variety of CMT phenotypic spectra, including CMT1F, CMT2E, dominant intermediate CMT G (CMTDIG), and autosomal recessive CMT [3].

However, the central nervous system (CNS) has been implicated in several CMT patients with GJB1 and MFN2 mutations [4,5], and CNS symptoms have been reported in CMT patients with NEFL mutations [6,7]. Cerebellar ataxia is one of the common symptoms of cerebellar dysfunction, which manifests as an inability to control or coordinate voluntary movements, with disturbance in planning and execution of movements [8]. Cerebellar ataxia has been reported in some *NEFL* patients [6,7,9,10,11]. From the literature review, among 173 patients with *NEFL* mutations, ataxia was found in 22 patients and cerebellar atrophy in 4 patients [6]. Even in patients who have been reported to have cerebellar ataxia, it is difficult to detect cerebellar atrophy using routine conventional brain MRI. Recently, diffusion tensor imaging (DTI) study has been widely used in the neuroscience field. DTI is employed to examine white matter microstructure, such as diffusion constraints that exist within the voxel; that is, at a spatial scale well below that at which the image acquisition resolution can be identified. Whereas the sensitivity of a conventional structural MRI depends on the spatial resolution used for imaging, the sensitivity of DTI relies on its diffusion measurement of water molecules on a microscopic scale (up to μm), irrespective of the spatial resolution used for imaging (up to mm). In hereditary ataxias, DTI study has shown to be useful, revealing white matter abnormalities, and this change is associated with the severity of ataxia [12,13,14,15]. We previously described the DTI abnormality of cerebral white matter (WM) that correlated with clinical disability in CMT patients with MFN2, GJB1, and NEFL mutations [10]. Additionally, there has been a report of a combined structural and diffusion MRI study of CMT1A patients [16]. However, there has been no report of volumetry or DTI studies of the cerebellum in patients with *NEFL* mutations. We employed DTI to assess changes in white matter microstructure by leveraging its sensitivity to diffusion measurement on a microscopic scale.

In this study, we used both volumetry and DTI to search for structural changes in the cerebella of 47 controls and 47 CMT patients with *PMP22*, *MFN2*, *GJB1*, and *NEFL* mutations. Interestingly, we observed significant volumetric changes in the cerebella of CMT patients, especially with *NEFL* mutations, which were associated with the presence of cerebellar ataxia in this genetic subgroup.

## 2. Materials and Methods

### 2.1. Participants

We enrolled 94 study participants, including 47 healthy controls and 47 CMT patients: 10 CMT1A patients with *PMP22* duplication, 15 CMT2A patients with *MFN2* mutations, 11 CMTX1 patients with *GJB1* mutations, and 11 CMT patients (3 CMT1F, 2 CMT2E, and 6 CMTDIG) with *NEFL* mutations (Table 1). Except one L312P *NEFL* patient with cerebellar atrophy, the cerebella showed normal findings in a routine conventional brain MRI. The determination of causative mutations and clinical assessments were performed as described previously [10].

### 2.2. Clinical Assessments

Clinical assessments were obtained by detailed history-taking and physical examination to assess muscle weakness, atrophy, sensory loss, and deep tendon reflexes. In order to determine physical disability, we used three scales: the Medical Research Council (MRC) scale for muscle strength [17], the functional disability scale (FDS) [18], and the CMT neuropathy score (CMTNS) [19]. Flexor and extensor muscle strength was evaluated using the standard MRC scale. Age at onset was determined by asking patients at what age their symptoms first appeared. Ataxia was scored by an experienced neurologist according to the scale for the assessment and rating of ataxia (SARA) while subjects performed each task. SARA is a clinical scale developed by Schmitz-Hübsch and colleagues [20] that assesses a range of different impairments in ataxia, ranging from speech to balance. The scale comprises 8 categories: gait (0–8 points), stance (0–6 points), sitting (0–4 points), speech disturbance (0–6 points), finger chase (0–4 points), nose–finger test (0–4 points), fast alternating hand movement (0–4 points), and heel–shin slide (0–4 points). Once the clinician assesses each of the 8 categories for an individual, they can further compute the cumulative score, ranging from 0 (no ataxia) to 40 (most severe ataxia), to determine the ataxic subject’s severity of ataxia.

### 2.3. MR Volumetry and Diffusion Tensor Imaging (DTI)

We performed 3-T MRI for volumetry and DTI with high resolution 3-dimensional T1-weighted images and diffusion-weighted EPI sequences (Philips Achieva v2.6, Best, The Netherlands). MRI processing consisted of AC-PC co-registration between T1-images, multimodal bias-correction, and applying a multi-label joint fusion algorithm. Using the BRAINSTools suite, the brain was divided into 217 sub-regions, which were then classified as either cerebral or cerebellar for further analysis. Finally, age and total intracranial volumes were adjusted to compare the WM and gray matter (GM) volumes. All volumetric data were processed through a fully automated procedure, BRAINS Auto-Workup (BAW) [21,22,23], improved with SyN registration from the Advanced Normalization Toolkit in the BRAINSTools suite (https://github.com/BRAINSia/BRAINSTools (accessed on 31 March 2021)) [23]. The resulting data set of bias-corrected average T1 images was subsequently segmented for subcortical structures using an automated segmentation framework, ANTs MALF [23,24]. Both the raw data and the resultant segmentation were rigorously reviewed for validity by visual inspection.

DTI data were collected using the same parameters (TR, 4500 ms; TE, 68 ms; EoV RL, 240 mm × AP 240 mm; FH, 135 mm; matrix size, 128 × 128 mm, 3 mm slice thickness, no gap; flip angle, 90 degrees; voxel size: RL, 2; AP, 2; 32 directions with b-value 1000 s/mm^2^, and one null image with *b*-value 0 sec/mm^2^; total diffusion gradient, 80 mT/m; NSA = 1; 45 slices in transverse plane) and analyzed using the tools included in the FSL (http://fsl.fmrib.ox.ac.uk/fsl/ (accessed on 31 March 2021)) to compute DTI-derived measures, including fractional anisotropy (FA), axial diffusivity (AD), and radial diffusivity (RD), as described elsewhere [10]. The protocol for this study was approved by the Institutional Review Board, and informed consent was obtained from all patients and from parents of patients younger than 18 years of age.

### 2.4. Statistical Analysis

We analyzed volumetric data using R software version 3.3. Whole cerebellar tissue and regional cerebellar volumes were standardized to the individual intracranial volume (ICV) to account for differences in head size. We performed all analysis of volume differences using % scale, volume per ICV in percentage [23]. Left- and right-side volumes of the cerebellum were combined. A comparison of cerebellar volumes between normal control and CMT patients for genetic subtypes was performed using Analysis of Variance (ANOVA), using age and sex as covariates. A Bonferroni correction was applied to adjust for multiple comparisons.

The tract-based spatial statistics (TBSS) approach was used for voxelwise statistical analysis, with correction for age and sex [10,25,26]. We compared the DTI-derived measures between control and CMT groups with genetic subtypes. The familywise error rate was controlled using the threshold-free cluster enhancement (TFCE) approach [26], by which cluster-like structures are enhanced without having to define clusters in a binary way. Group differences in DTI from TBSS were evaluated with TFCE-corrected *p* < 0.05 to adjust for multiple comparisons. The FA and diffusivity measures of DTI were tested for correlation with clinical variables by using Spearman’s rank correlation coefficients (ρ) [10,25].

## 3. Results

The clinical features and nerve conduction studies of the 47 CMT patients are shown in Table 1. CMT patients had muscle weakness and atrophy, predominantly in the distal legs, ranging from mild weakness to complete paralysis. Genetic mutations included eight *MFN2* mutations, seven *GJB1* mutations, and six *NEFL* mutations. CMT patients with *NEFL* and *GJB1* mutations exhibited diverse cerebellar involvements, including cerebellar ataxia, dysarthria, dyssynergia, and dysmetria. In particular, the frequency of the cerebellar ataxia was high in patients with *NEFL* mutations (72.7%), compared to *GJB1* mutations (9.1%), *PMP22* duplication (0%), and *MFN2* mutations (0%).

**Figure 1:**
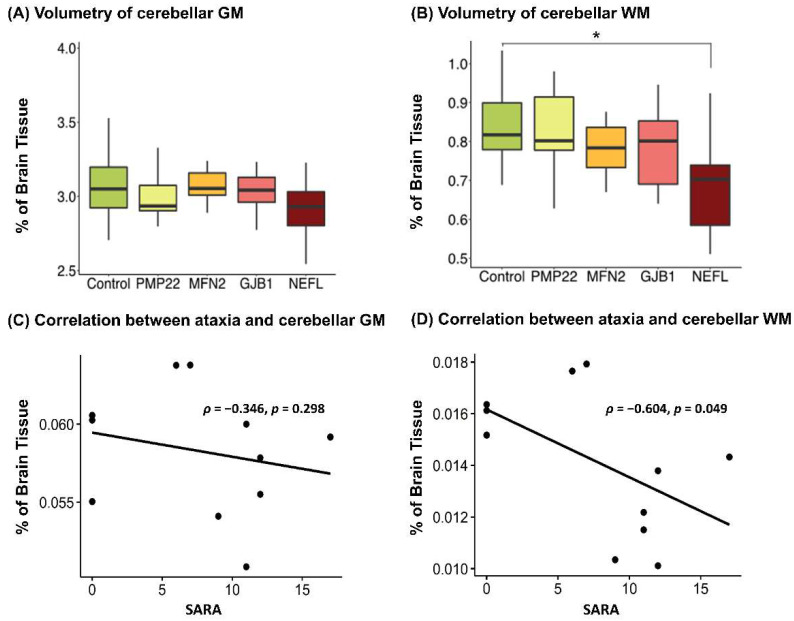
Volumetric findings of gray matter (**A**) and white matter (**B**) in cerebellum of CMT patients with different genetic subtypes. CMT patients with *NEFL* genetic mutations demonstrate distinct decreased volumetric changes in cerebellar white matter (**B**) but not cerebellar gray matter (**A**), while neither cerebellar GM nor WM changes were observed in the subgroups with *PMP22* duplication, *MFN2*, or *GJB1* mutations. (**C**) Correlation between SARA and cerebellar GM in *NEFL* subgroup. (**D**) Correlation between SARA and cerebellar WM in *NEFL* subgroup. Significant difference in paired test was marked with *, *p* < 0.05. Abbreviations: GM, gray matter; WM, white matter.

The mean volume proportion of the cerebellar region was 3.08% (±0.25, V/ICV) for normal controls, while most of the CMT groups showed lower cerebellar regional volumes (Figure 1). A group comparison showed a significant difference in cerebellar GM and WM volumes in the *NEFL* mutation group (Figure 1A,B). The difference in cerebellar WM in the *NEFL* subgroup, compared with the control group, remained significant, even after multiple comparison adjustments (−0.151 ± 0.031, *p* = 0.0083, Figure 1B). The cerebellar WM volume of patients in the *MFN2* group revealed a nonsignificant tendency to be reduced (−0.051 ± 0.028, *p* = 0.07). However, there was no statistically significant cerebellar atrophy in the other genotypes (*PMP22* duplication, *MFN2*, or *GJB1* mutation). Among the *NEFL* subgroup, SARA showed a negative correlation with the volume proportion of cerebellar WM (Spearman’s ρ = −0.604, *p* = 0.049) but not with cerebellar GM (Spearman’s ρ = −0.346, *p* = 0.298).

**Figure 2:**
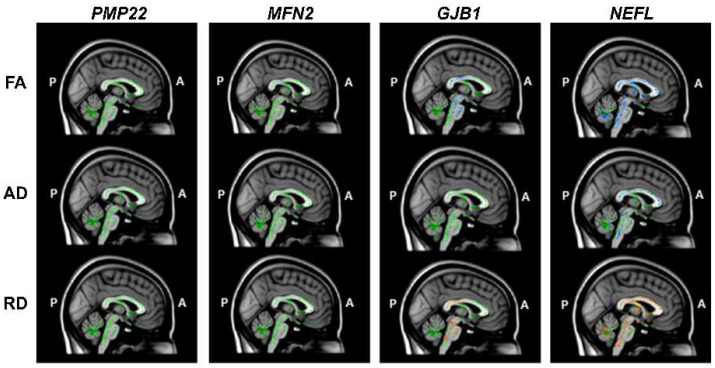
DTI findings in CMT patients with different genetic subtypes. DTI abnormalities, including reduced FA and AD and increased RD values, were observed mostly in the genetic subgroup with *NEFL* mutations. Decreased values depicted in blue, and increased values in red, with corrected *p* < 0.05.

**Figure 3:**
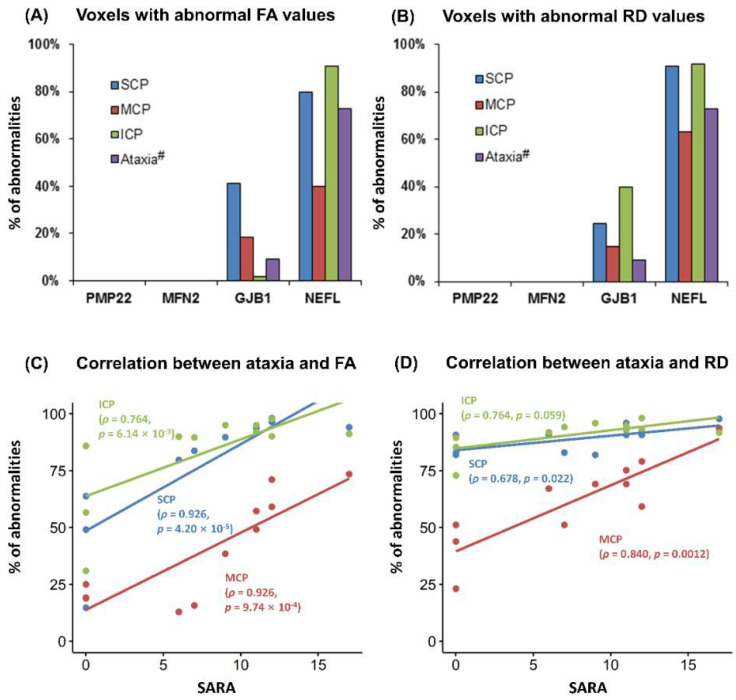
DTI abnormalities of FA and RD in different genetic subtypes of CMT with ataxia as a neurologic manifestation. Reduced FA (**A**) and increased RD (**B**) in major cerebellar WM tracts, including the superior cerebellar peduncle (SCP), the middle cerebellar peduncle (MCP), and the inferior cerebellar peduncle (ICP), were observed, predominantly in the *NEFL* subgroup, but also subtle abnormalities in the *GJB1* subgroup. Interestingly, higher scores on SARA correlated with more severe abnormalities in FA (**C**) and RD values (**D**) in DTI of patients with *NEFL* mutation.

DTI of the cerebellum revealed significantly reduced FA and AD and increased RD values, most prominently in the *NEFL* genetic subgroup and mildly in *GJB1* subtype, but no changes in the other subgroups with *PMP22* duplication or *MFN2* mutation (Figure 2). In the *NEFL* genetic subtype, DTI of the cerebellum revealed significantly reduced FA values in 90.9% of the voxels of the superior cerebellar peduncle (SCP), 63.4% of the middle cerebellar peduncle (MCP) voxels, and 92.0% of the inferior cerebellar peduncle (ICP) voxels (Figure 3A). Similarly, significantly deceased AD values were found in 68.0%, 57.0%, and 57.4% of the voxels, and increased RD values in 79.7%, 40.1%, and 90.9% of the voxels in the SCP, the MCP, and the ICP, respectively (Figure 3B). In the case of *GJB1* mutation, reduced FA values were found in 24.3%, 14.9%, and 0.4% of the voxels, and increased RD values in 41.4%, 18.3%, and 1.9% of the voxels of the SCP, the MCP, and the ICP, respectively. AD values were not different from those of healthy controls. Interestingly, manifestation of cerebellar ataxia was largely restricted to the *NEFL* subgroup; it was observed clinically in 8 out of 11 CMT patients in the *NEFL* subgroup (72.7%) but in only 1 out of 11 CMT patients (9.1%) in the *GJB1* subgroup (Figure 3A,B). Among the *NEFL* subgroup, SARA was correlated to voxels with decreased AD values of the SCP (Spearman’s ρ = 0.926, *p* < 0.01), the MCP (Spearman’s ρ = 0.848, *p* < 0.01), and the ICP (Spearman’s ρ = 0.764, *p* < 0.01). Similarly, SARA was correlated to voxels with increased RD values of the SCP (Spearman’s ρ = 0.678, *p* = 0.02) and the MCP (Spearman’s ρ = 0.840, *p* < 0.01). Thus, abnormal DTI findings in cerebellar WM tracts, combined with reduced cerebellar WM volume, occurred predominantly in the *NEFL* subgroup, the subtype associated with cerebellar ataxia symptoms.

## 4. Discussion

In this study, we found structural evidence of cerebellar WM abnormalities, including reduced cerebellar WM by volumetry in the *NEFL* mutation group and microstructural abnormalities by DTI of the SCP, MCP, and ICP in the *NEFL* and *GJB1* subgroups. It has been reported that brain T1-weighted MR images in CMT patients with the N98S *NEFL* mutation showed cerebellar atrophy [26]. It was not clear, however, whether cerebellar atrophy could be a common feature seen in the *NEFL* mutation in the previous studies. We examined the MRI volumetry and DTI studies in CMT patients, including *NEFL* mutations harboring various mutation sites. As a result, we confirmed that cerebellar atrophy is a common phenomenon observed in the *NEFL* mutation group. In our study, we proved that *NEFL* mutations affect the WM of the cerebellum but not the GM. In addition, cerebellar ataxia, which is commonly manifested in patients with *NEFL* mutations, is not well observed in the other genetic mutations, such as *PMP22* and *MFN2*, except in 1 out of 11 patients with *GJB1* mutations. To our knowledge, this is the first report of volumetric and DTI study of CMT patients with *NEFL* mutations showing cerebellar WM abnormality.

According to the literature review, in 173 patients with *NEFL* mutations, ataxia was found in 22 patients and cerebellar atrophy in 4 patients [6,7]. This means that some *NEFL* patients showed only cerebellar ataxia without cerebellar atrophy. In the present study, cerebellar ataxia was found in 72.7% of the *NEFL* subgroup, but cerebellar atrophy was observed in only one patient with the L312P *NEFL* mutation. This discrepancy may be due to the fact that ataxia caused by cerebellar dysfunction is an earlier symptom than cerebellar atrophy. Therefore, it is difficult to find cerebellar atrophy with a routine conventional brain MRI, and cerebellar involvements in *NEFL* patients may be underestimated. Interestingly, reduced FA and increased RD were detected in a high proportion of voxels in the SCP, MCP, and ICP in the *NEFL* mutation group. Furthermore, these DTI findings showed correlation with severity of cerebellar ataxia in this group. The MCP contains pontocerebellar fibers, which receive signals from corticopontine fibers, and the cerebellum, in turn, projects to the ventrolateral nucleus of the thalamus, which projects to the motor cortex to form a circuit. The ICP contains the posterior spinocerebellar tract, which relays unconscious proprioception, as well as the vestibulocerebellar tract. Thus, ataxia in the *NEFL* subgroup could be explained by the involvement of the SCP, MCP, and ICP.

Additionally, we found DTI cerebellar abnormalities in the *GJB1* mutation group. One patient with the C179X *GJB1* gene mutation showed cerebellar ataxia without cerebellar atrophy in routine conventional brain MRI. DTI of the cerebellum showed moderately reduced FA values in 24.3% of the voxels of the SCP in *GJB1* group. However, it was not easy to tell whether the patient with ataxia had distinctive DTI features because there was only one patient who had relatively mild ataxia (SARA 7).

Nevertheless, MRI volumetry or DTI can be helpful for early detection of cerebellar dysfunction in CMT patients with *NEFL* and/or *GJB1* mutations, especially in cases harboring cerebellar ataxia. In fact, *GJB1* has been reported to relate to mitochondrial function in motor neurons in CNS [27] and to be expressed in both Schwann cells and oligodendrocytes, the myelinating glia of the PNS and CNS, respectively [28].

Our findings provide structural evidence for cerebellar WM involvements in CMT patients with *NEFL* mutations investigated by MRI volumetry and DTI studies, which is related to the prevalent manifestation of cerebellar ataxia in this genetic subtype. This study also demonstrates that quantitative MRI, such as volumetry and DTI, can be useful for clinical characterization, including future development of CNS involvement in CMT patients with diverse genetic abnormalities.

## Figures and Tables

**Table 1:** Basic demographic and clinical characteristics of study population.

	Normal Control	*PMP22* Duplication	*MFN2* Mutations	*GJB1* Mutations	*NEFL* Mutations
Number	47	10	15	11	11
Female (%)	49	45	60	45	36
Age at exam (years)	37.5 ± 14.6	42.0 ± 14.7	31.9 ± 13.6	39.4 ± 16.5	39.5 ± 11.8
Age at onset (years)	-	11.2 ± 6.9	10.6 ± 11.3	23.5 ± 13.7	14.0 ± 4.5
Muscle weakness	No	UL < LL ^a^	UL < LL	UL < LL	UL < LL
Sensory loss	No	Yes	Yes	Yes	Yes
MRC ^b^ (arm)	5	3.9 ± 0.3	1.6 ± 1.7	3.8 ± 0.9	2.1 ± 0.7
MRC ^c^ (leg)	5	2.1 ± 1.2	1.4 ± 1.3	3.5 ± 1.3	1.8 ± 0.6
FDS ^d^	0	2.0 ± 1.1	3.9 ± 2.2	2.3 ± 1.0	3.0 ± 1.0
CMTNS v2 ^e^	0	14.3 ± 5.5	18.7 ± 8.9	12.6 ± 6.3	14.7 ± 5.7
Cerebellar ataxia	0	0	0	1 (9.1%)	8 (72.7%)
SARA ^f^	0	0	0	7.0	10.6 ± 3.4
Peripheral ulnar nerve conduction studies					
CMAP ^g^ (mV)	15.9 ± 2.8	6.6 ± 3.5	7.2 ± 6.0	9.3 ± 3.0	5.8 ± 4.8
MNCV ^h^ (m/s)	61.2 ± 3.2	19.6 ± 3.9	52.3 ± 8.4	43.3 ± 9.0	36.0 ± 9.0
SNAP ^i^ (uV)	23.1 ± 7.2	5.2 ± 1.7	8.4 ± 4.8	5.8 ± 3.3	4.3 ± 2.1
SNCV ^j^ (m/s)	51.6 ± 2.5	21.3 ± 4.8	32.9 ± 5.8	30.5 ± 3.2	36.3 ± 3.8

^a^ UL < LL, upper limb weakness < lower limb weakness; ^b^ MRC (arm), Medical Research Council scale for motor weakness from finger abduction; ^c^ MRC (leg), Medical Research Council scale for motor weakness from ankle dorsiflexion; ^d^ FDS, functional disability scale; ^e^ CMTNS v2, Charcot–Marie–Tooth neuropathy score version 2; ^f^ SARA scale is made up of 8 categories with scores ranging as, gait (0–8 points), stance (0–6 points), sitting (0–4 points), speech disturbance (0–6 points), finger chase (0–4 points), nose–finger test (0–4 points), fast alternating hand movement (0–4 points), heel–shin slide (0–4 points); ^g^ CMAP, compound muscle action potential; ^h^ MNCV, motor nerve conduction velocity; ^i^ SNAP, sensory nerve action potential; ^j^ SNCV, sensory nerve conduction velocity. Normal NCV values: ulnar motor nerve, ≥51.1 m/s; ulnar sensory nerve, ≥37.5 m/s. Normal amplitude values: ulnar motor nerve, ≥8 mV; ulnar sensory nerve, ≥7.9 uV.

## Data Availability

All of the raw and processed data for genetic and imaging analysis were stored and can be accessed in our laboratory, which is supervised by the corresponding authors, B.-O.C. and H.W.L., respectively.
